# Identification of candidate genes associated with clinical onset of Alzheimer’s disease

**DOI:** 10.3389/fnins.2022.1060111

**Published:** 2022-12-20

**Authors:** Wang Liao, Haoyu Luo, Yuting Ruan, Yingren Mai, Chongxu Liu, Jiawei Chen, Shaoqing Yang, Aiguo Xuan, Jun Liu

**Affiliations:** ^1^Department of Neurology, The Second Affiliated Hospital, Guangzhou Medical University, Guangzhou, China; ^2^Department of Rehabilitation, The Second Affiliated Hospital, Guangzhou Medical University, Guangzhou, China; ^3^Guangzhou Medical University, Guangzhou, China; ^4^School of Basic Medical Sciences, Guangzhou Medical University, Guangzhou, China

**Keywords:** Alzheimer’s disease, genes, transcriptomic analysis, GEO, dementia

## Abstract

**Background and objective:**

Alzheimer’s disease (AD) is the most common type of dementia, with its pathology like beta-amyloid and phosphorylated tau beginning several years before the clinical onset. The aim is to identify genetic risk factors associated with the onset of AD.

**Methods:**

We collected three microarray data of post-mortem brains of AD patients and the healthy from the GEO database and screened differentially expressed genes between AD and healthy control. GO/KEGG analysis was applied to identify AD-related pathways. Then we distinguished differential expressed genes between symptomatic and asymptomatic AD. Feature importance with logistic regression analysis is adopted to identify the most critical genes with symptomatic AD.

**Results:**

Data was collected from three datasets, including 184 AD patients and 132 healthy controls. We found 66 genes to be differently expressed between AD and the control. The pathway enriched in the process of exocytosis, synapse, and metabolism and identified 19 candidate genes, four of which (VSNL1, RTN1, FGF12, and ENC1) are vital.

**Conclusion:**

VSNL1, RTN1, FGF12, and ENC1 may be the essential genes that progress asymptomatic AD to symptomatic AD. Moreover, they may serve as genetic risk factors to identify high-risk individuals showing an earlier onset of AD.

## 1 Introduction

Alzheimer’s disease (AD) is a chronic degenerative disease characterized by the extracellular deposition of beta-amyloid and intracellular accumulation of phosphorylated tau protein (Spina et al., 2021). For older adults, AD is the most common cause of dementia, with an incidence rate of approximately 1.5% among people over 65 years old and nearly 50% among people over 90 years old (Scheltens et al., 2021). Interestingly, cognitive impairment of many AD patients occurs substantial years after pathological changes, such as amyloid and neurofibrillary tangles (NFTs). In contrast, more than 20% of the aged population have an amyloid deposition. Individuals with intact cognition and neuropathology consistent with AD were called asymptomatic AD (AsymAD) (Patel et al., 2019). AsymAD is distinguishable from normal aging based on neuropathology, brain imaging, and cerebrospinal fluid biomarkers (Ayodele et al., 2021). While some of these individuals progress to symptoms related to cognition, which deviate from mild cognitive impairment (MCI), and then to AD, not all do. They are, therefore, a heterogeneous group, representing those with prodromal AD and those impervious to AD despite having pathological hallmarks. These individuals are highly likely to develop symptomatic AD. Abundant evidence has demonstrated genetic risk factors of AD, such as APP, PSEN1, PSEN2, and apolipoprotein ε4 allele (APOE4) (Lefterov et al., 2019). However, transcriptomic changes in the brain, which might reveal AD vulnerability, are currently unknown. In the present study, we identified genetic risk factors for AD onset in asymptomatic and symptomatic individuals with clear signs of AD pathology at autopsy. Understanding the fundamental changes may shed light on specific biological mechanisms involved in early pathological hallmarks of AD, providing new therapeutic targets for early intervention.

## 2 Materials and methods

### 2.1 Data acquisition and processing

We searched the microarray sequencing datasets of AD patients’ post-mortem brain samples for 5 years on the Gene Expression Omnibus database (GEO). Then we removed the datasets with incomplete annotation of platform annotation information. Finally, we selected three datasets of the same platform [GSE139384 (Morimoto et al., 2020), GSE118553 (Patel et al., 2019), and GSE132903 (Piras et al., 2019)]. We integrated the temporal lobe data in the post-mortem brains of 184 AD patients and 132 healthy people with ComBat package (Supplementary Data Sheet 1). All the above datasets were gathered using the Illumina HumanHT-12 V4.0 platform.

### 2.2 Screening of key differentially expressed genes

We screened the differentially expressed genes (DEGs) between AD patients and control in the processed new dataset using the limma R package. We first use the lmFit function to construct the linear model and then use the contrasts fit function to calculate the contrast of the model (estimated coefficient and standard error). Next, eBayes function to compute moderated t-statistic, moderated F-statistic, and log-odds of differential expression, and use the top table function (adjust.method is ‘ fdr ’) to extract the gene table. Finally, the differential genes were screened by the standard of |logFoldChange| = 0.5, adjust *P* = 0.05, and displayed visually by ggplot2 and heatmap. Further, we performed weighted correlation network analysis (WGCNA) with the new dataset we got to identify the co-expression of hub genes.

### 2.3 Functional enrichment and pathway analysis of DEGs

All the DEGs were analyzed by GO (Gene ontology) and KEGG (Kyoto Encyclopedia of Genes and Genomes) enrichment analysis to investigate the biological mechanisms. With enrichgo and enrichKEGG functions in the clusterProfiler package, we analyzed the differential genes and logFC values to obtain significantly enriched gene functions and pathways. GO analysis was annotated by org.Hs.eg.db package, KEGG analysis set hsa as species information, *p* value and *q* value were set to 0.05, and visualize with barplot and GOChord. In order to reduce the poor enrichment results caused by the fixed threshold method for screening differential genes, we further performed GSEA analysis on the overall genes list with logFC value of the new dataset using the gseKEGG function in the clusterProfiler package, organism is hsa.

### 2.4 Establishment of PPI network

We input DEGs list into the String database^1^ and obtained a protein interaction network diagram and data forms. Then, we downloaded the data tables and imported them into cytoscape software, using the cytohubba plug-in to calculate the key nodes. Based on the results of MCC algorithm, the interactive network diagrams of different colors are drawn according to the calculated scores.

### 2.5 Differential genes between symptomatic and asymptomatic AD

Differentially expressed genes were first analyzed between asymptomatic and symptomatic AD groups in the GSE118553 dataset to identify the genetic risk factors for symptomatic AD. We then overlapped these DEGs with AD-related DEGs derived from WGCNA. Feature importance with a logistic regression analysis were used to identify the predicted importance of the DEGs further. Specifically, we first sorted out the expression of immune-related differential expressed genes in the validation set. Next, the matrix was input into SPSS 26.0 software, and the binary logistic regression analysis with the forward conditional method was carried out with whether it was symptomatic AD as the dependent variable and the expression of each gene as the independent variable. Finally, the matrix of the key genes successfully incorporated into the model was input into the R language, the rms package was loaded, and the linear regression model between these genes and symptomatic AD was constructed using the lrm function. Then, the nomogram function was used to draw a nomogram representing the regression fitting to visually demonstrate the predictive power of these genes.

## 3 Results

### 3.1 Demographic characteristics and data processing

A total of 184 AD patients and 132 normal controls were included in this study. The demographic information is shown in Tables 1, 2. After standardized processing and removing the batch effect, the distribution of the three datasets tends to be comparable, indicating that the processed data is highly uniform and quality (Supplementary Figure 1).

**TABLE 1:** Datasets characteristics.

Datasets	Tittle	Platform	Organism	Submission date	PMID	Samples
GSE139384	Synaptopathy in Kii ALS/PDC, a disease concept based on transcriptome analyses of human brains	GPL10558 (Illumina HumanHT-12 V4.0 expression beadchip)	*Homo sapiens*	October 25, 2019	32422904	6
GSE118553	Transcriptomic analysis of probable asymptomatic and symptomatic Alzheimer’s brains			August 14, 2018	31063847	115
GSE132903	Transcriptome changes in the Alzheimer’s middle temporal gyrus: importance of RNA metabolism and mitochondria-associated membrane (MAM) genes			June 18, 2019	31256118	195

**TABLE 2:** Datasets clinical characteristics.

	Group		Gender		Age	
	AD	Control	Male	Female	≤85 years	>85 years
GSE139384	3	3	6	0	4	2
GSE118553	84	31	64	65	65	50
GSE132903	97	98	99	96	98	97

### 3.2 Identification of DEGs between AD patients and healthy

**FIGURE 1:**
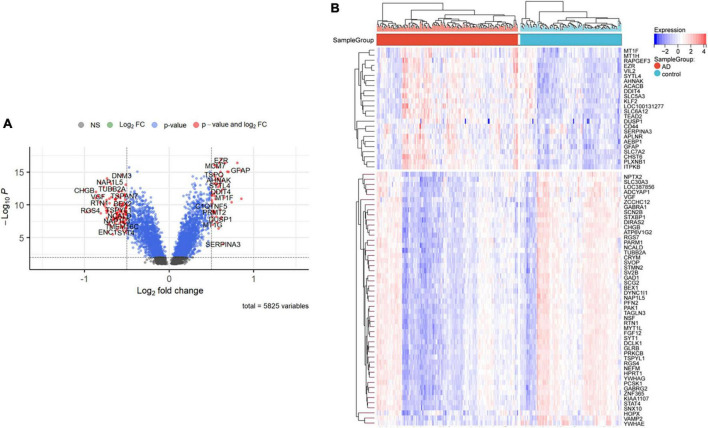
Differentially expressed genes between AD and control samples. **(A)** The right red genes represent significantly high expression in AD; the left red genes represent significantly high expression in control. **(B)** The heatmap shows the top 50 genes significantly expressed in AD or ND samples.

**FIGURE 2:**
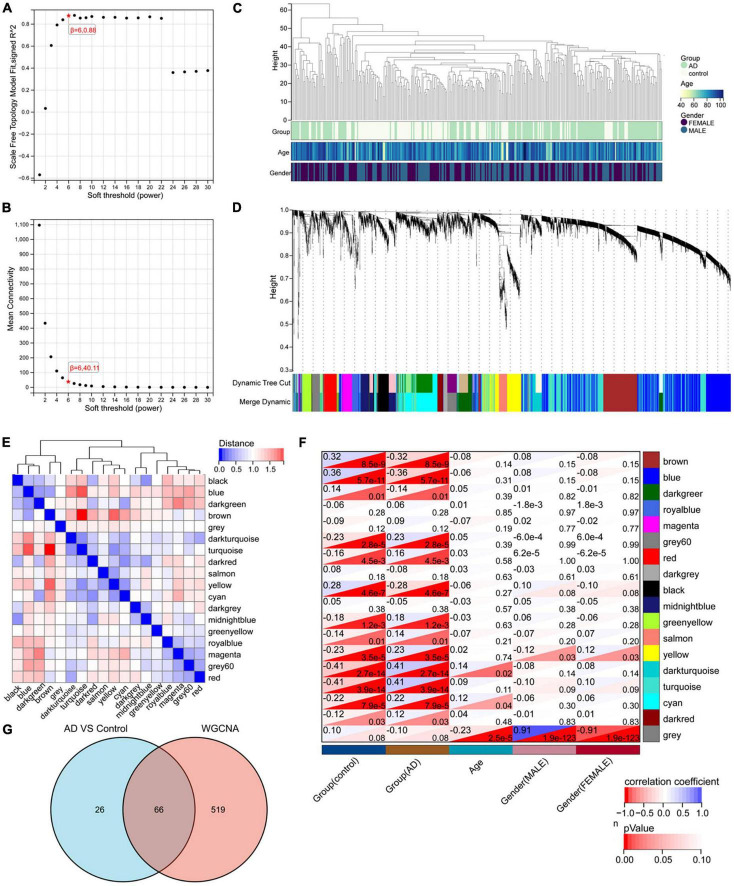
WGCNA analysis results. **(A)** The corresponding scale-free topological model fit indices at different soft threshold powers. **(B)** The corresponding mean connectivity values at other soft threshold powers. **(C)** Sample clustering. **(D)** Cluster dendrogram of genes. **(E)** Correlation map of vector clustering of 18 modules. **(F)** Correlation heatmap between modules and clinical features, the lower left corner of each grid is the *P*-value, and the upper right corner is the correlation coefficient. **(G)** The Wayne diagram of intersection with 585 hub genes obtained by WGCNA and 92 DEGs by limma.

Nighty-two DEGs were found, including 68 down-regulated and 24 upregulated genes (Figures 1A,B). Next, we got the Median Absolute Deviation (MAD) of each gene, and the most minor top 30% genes were removed. Then we removed outlier genes and samples using the goodSampleGenes method in the R software package WGCNA (Figure 2C) and constructed a scale-free co-expression network. Then we evaluated the optimal power using the pick Soft Threshold function in the WGCNA package with a threshold of 0.9 (Figure 2A). As is shown in Figure 2B, the weighted gene network constructed with a power value of six has good connectivity. To classify genes with similar expression profiles into Gene modules, we set the gene tree’s minimum size as 30, the sensitivity as 3, and the distance less than 0.25 to synthesize modules. Finally, 18 co-expression modules were obtained, and gene clustering maps and module vector clustering maps were shown in Figures 2D,E (the gray module is considered a gene set that cannot be assigned to any module). Next, clinical information of the dataset was included to clarify its correlation with the modules (Figure 2F). Finally, based on the truncation criteria with |MM| > 0.8 and |GS| > 0.2, we selected the top five modules with the highest coefficient as the key modules: turquoise, dark turquoise, blue, brown, and black modules. Among them, 585 genes with high connectivity were extracted as hub genes, intersecting with the previously obtained 92 DEGs. A total of 66 co-expressed differential genes were left (Figure 2G).

### 3.3 Enrichment of DEGs and protein interaction network

**FIGURE 3:**
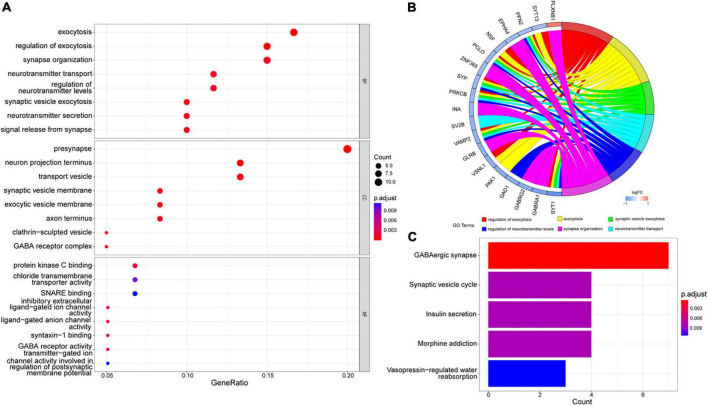
GO and KEGG enrichment analysis. **(A)** The most significant enrichment pathways in the GO database. **(B)** Chord diagram of GO enrichment analysis. **(C)** The most considerable enrichment pathways in the KEGG database.

We analyzed these 66 DEGs in GO and KEGG databases. We found that the GO database enriched regulating exocytosis, synapse organization, presynapse, neuron projection terminus, and transport vesicle pathways. Moreover, it is enriched in protein kinase C binding, chloride transmembrane transporter activity, and SNARE binding pathways (Figure 3A). The GO plot package chord diagram showed that SYT13, PFN2, NSF, PCLO, SYP, PRKCB, VAMP2, VSNL1, and SYT1 were significantly enriched in multiple major pathways (Figure 3B). While in the KEGG database is enriched in GABAergic synapse, synaptic vesicle cycle, insulin secretion, morphine addiction, and vasopressin-regulated water reabsorption pathways (Figure 3C). To discuss the pathway enrichment of the whole sample more comprehensively, we further performed GSEA analysis. The results showed that these genes were significantly enriched in 21 pathways, especially those associated with Alzheimer’s disease and neurodegenerative diseases (Table 3).

**TABLE 3:** GSEA results.

ID	Description	setSize	Enrichment score	Adj. *P*-value
hsa05168	Herpes simplex virus 1 infection	467	0.284718529	8.72 × 10^–5^
hsa05022	Pathways of neurodegeneration	434	−0.333872677	9.04 × 10^–5^
hsa05010	Alzheimer’s disease	345	−0.318194061	3.88 × 10^–3^
hsa05014	Amyotrophic lateral sclerosis	320	−0.335493776	9.26 × 10^–4^
hsa05016	Huntington disease	269	−0.363160142	3.04 × 10^–4^
hsa05020	Prion disease	240	−0.398580749	3.67 × 10^–5^
hsa05012	Parkinson’s disease	232	−0.41709762	4.81 × 10^–5^
hsa04024	cAMP signaling pathway	217	−0.315166868	4.24 × 10^–2^
hsa03010	Ribosome	131	−0.360481409	4.24 × 10^–2^
hsa00190	Oxidative phosphorylation	103	−0.505392372	2.21 × 10^–5^
hsa04350	TGF-beta signaling pathway	92	0.417938926	1.33 × 10^–3^
hsa04911	Insulin secretion	86	−0.432626299	1.24 × 10^–2^
hsa04512	ECM-receptor interaction	86	0.363314004	4.22 × 10^–2^
hsa04260	Cardiac muscle contraction	81	−0.4123577	3.67 × 10^–2^
hsa04721	Synaptic vesicle cycle	78	−0.562303369	4.81 × 10^–6^
hsa05120	Epithelial cell signaling in Helicobacter pylori infection	68	−0.44664597	2.13 × 10^–2^
hsa05110	Vibrio cholerae infection	49	−0.547099768	1.48 × 10^–3^
hsa00620	Pyruvate metabolism	46	−0.496786058	2.67 × 10^–2^
hsa03050	Proteasome	45	−0.530909935	4.32 × 10^–3^
hsa00020	Citrate cycle (TCA cycle)	30	−0.6441294	1.17 × 10^–3^
hsa04966	Collecting duct acid secretion	27	−0.577941421	2.69 × 10^–2^

**FIGURE 4:**
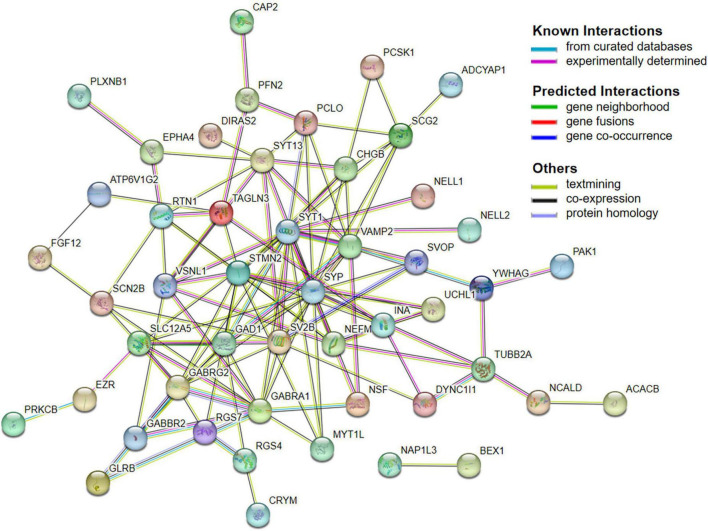
PPI network.

Next, 66 DEGs were further analyzed with STRING (Figure 4). After excluding 17 independent DEGs, we finally constructed a protein interaction network map according to the Maximal Clique Centrality (MCC) score, with the remaining 49 genes processed by Cytohubba. The top 10 hub genes are SYP, SYT1, GABRG2, GABRA1, SLC12A5, GAD1, SV2B, STMN2, VAMP2, and SCG2 (Table 4).

**TABLE 4:** Cytohubba results.

Gene name	Description	MCC	Degree	LogFC
SYP	Synaptophysin	398	18	−0.624723213
SYT1	Synaptotagmin-1	368	18	−0.972911963
GABRG2	Gamma-aminobutyric acid type B receptor subunit 2	280	11	−0.745760764
GABRA1	Gamma-aminobutyric acid receptor subunit alpha-1	266	12	−0.837374053
SLC12A5	Solute carrier family 12 member 5	241	7	−0.664705232
GAD1	Glutamate decarboxylase 1	157	9	−0.743098858
SV2B	Synaptic vesicle glycoprotein 2B	145	10	−0.665987783
STMN2	Stathmin-2	88	13	−0.640119418
VAMP2	Vesicle-associated membrane protein 2	81	10	−0.682915931
SCG2	Secretogranin-2	51	7	−0.665987783

### 3.4 Gene expression profiling of symptomatic and asymptomatic AD

**FIGURE 5:**
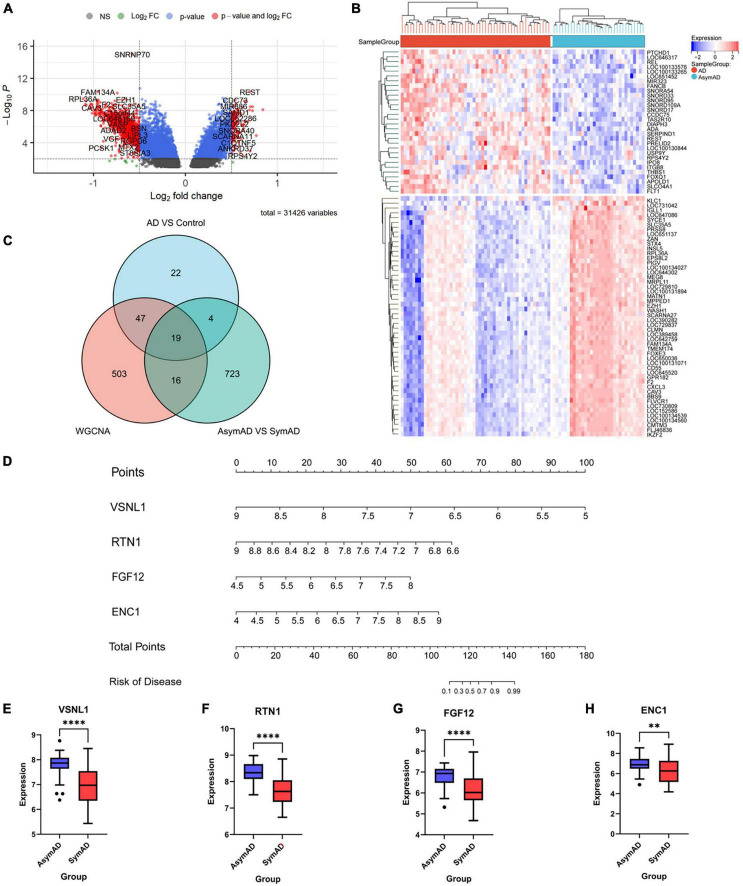
The gene expression difference between AsymAD and SymAD. **(A)** The right red genes represent significantly high expression in SymAD; the left red genes represent increased expression in AsymAD. **(B)** The heatmap shows the top 50 genes predominantly expressed in AsymAD or SymAD. **(C)** Wayne diagram to intersection DEGs of three difference analysis. **(D)** Nomogram of four essential genes. **(E–H)** Expression differences of four critical genes in AsymAD and SymAD group. ** means *p*-value < 0.01; **** means *p*-value < 0.0001.

Firstly, we extracted the temporal lobe information from AsymAD (*n* = 32) and SymAD (*n* = 52) in GSE118553 dataset. Seven hundred and sixty-two DEGs were identified, including 30 upregulated genes and 732 down-regulated genes (Figures 5A,B). Then, we sequence overlapped these DEGs with 92 AD-specific DEGs and WGCNA hub genes and finally obtained 19 key genes, including VSNL1, TAGLN3, SYP, SVOP, SLC12A5, RTN1, PCSK1, PAK1, NSF, NEFM, NCALD, LOC387856, HPRT1, GABRG2, FGF12, ENC1, CHGB, CAP2, and ADCYAP1 (Figure 5C and Table 5).

**TABLE 5:** Nineteen DEGs list.

Gene name	Description	LogFC	Adj. *P*-value
PCSK1	Phosphoenolpyruvate carboxykinase 1	−0.914236026	4.27 × 10^–4^
CAP2	Cyclase associated actin cytoskeleton regulatory protein 2	−0.8927109	6.52 × 10^–6^
LOC387856	Coiled-coil domain containing 184 (CCDC184)	−0.872134325	1.49 × 10^–6^
VSNL1	Visinin like 1	−0.823806102	2.71 × 10^–6^
PAK1	p21 (RAC1) activated kinase 1	−0.752922248	3.19 × 10^–6^
GABRG2	Gamma-aminobutyric acid type A receptor subunit gamma 2	−0.751904972	9.59 × 10^–6^
SLC12A5	Solute carrier family 12 member 5	−0.73219574	3.82 × 10^–6^
ADCYAP1	Adenylate cyclase activating polypeptide 1	−0.716567516	1.08 × 10^–3^
TAGLN3	Transgelin 3 (Neuronal protein NP2)	−0.715251481	2.70 × 10^–6^
NCALD	Neurocalcin delta	−0.694917396	8.29 × 10^–3^
SVOP	SV2 related protein	−0.666215273	1.93 × 10^–4^
RTN1	Reticulon 1 (Neuroendocrine-specific protein)	−0.663450406	6.98 × 10^–6^
FGF12	Fibroblast growth factor 12	−0.657390284	1.46 × 10^–4^
ENC1	Ectodermal-neural cortex 1	−0.649388586	2.01 × 10^–2^
CHGB	Chromogranin B	−0.645099164	5.03 × 10^–6^
NSF	*N*-Ethylmaleimide sensitive factor, vesicle fusing ATPase	−0.62336505	4.38 × 10^–6^
NEFM	Neurofilament medium chain	−0.609879042	2.56 × 10^–3^
SYP	Synaptophysin	−0.599413737	4.34 × 10^–6^
HPRT1	Hypoxanthine phosphoribosyltransferase 1	−0.587576219	7.57 × 10^–6^

### 3.5 VSNL1, RTN1, FGF12, and ENC1 might be genetic risk factors associated with AD onset

Next, we performed a stepwise logistic regression analysis of these 19 DEGs to identify symptomatic AD risk factors and obtained four genes: VSNL1, RTN1, FGF12, and ENC1 (Table 6). Specifically, the goodness of fit and predictive ability of the model are as follows: (1) according to the Omnibus Test results, the chi-square value of the model is 54.070, and the *p*-value is <0.001, indicating that the model is statistically significant; (2) according to the results of Hosmer and Lemeshow Test, the *p*-value of the fitting model is 0.229 > 0.05, which indicates that the information in the current data has been fully extracted and the goodness of fit of the model is high; (3) according to the results of the classification table, the sensitivity of the model is 90.4% and the specificity is 80.4%. On the whole, it has a correct prediction rate of 88.1% for all samples, indicating that it has good predictive ability. Through the nomogram, we found that decreased VSNL1, RTN1, and increased FGF12 and ENC1 were positively correlated with disease risk (Figure 5D). Then, we further examined the expression levels of these four genes in disease grouping and observed that the expression of all these four genes was significantly lower in the SymAD group (Figures 5E–H).

**TABLE 6:** Logistic regression analysis of four key genes.

Variable	*B*	Std. err.	Wald	*P*	OR	95% CI
VSNL1	−5.058	1.588	10.147	0.001	0.006	0.000–0.143
RTN1	−5.208	1.768	8.682	0.003	0.005	0.000–0.175
FGF12	2.884	1.495	3.721	0.054	17.891	0.955–335.245
ENC1	2.347	0.851	7.607	0.006	10.458	1.973–55.444

## 4 Discussion

The present study analyzed three datasets of post-mortem brain samples of AD patients and the healthy. Since the temporal lobe is demonstrated to be most related to AD, we extract the specific brain region for further analysis. A dataset with information on asymptomatic AD was applied for subsequent analysis of genetic risk factors and their onset. Differential analysis, WGCNA analysis, function enrichment pathway analysis, protein interaction network analysis, and logistic regression analysis were used to interpret the data. Finally, we found four essential genes closely related to AD, including VSNL1, RTN1, FGF12, and ENC1.

A lot of analytical methods have found many risk molecules associated with AD (Talwar et al., 2014). However, most recent studies still focus on genetic characteristics of early-onset or familial AD (Mold et al., 2020) and pay less attention to the gene expression of late-onset AD. Therefore, we firstly investigated the differential gene expression profiling between AD and the healthy. Among the 92 candidate genes, the most significantly upregulated genes were APLNR, GFAP, and AEBP1, while the most down-regulated genes were CHGB, SYT1, and RGS4. With further analysis by WGCNA, we finally obtained 66 differentially expressed genes.

Moreover, SYP and SYT1 were identified as top hub gene nodes in the protein interactions network, suggesting that these two genes play a vital role in the pathogenesis of AD. SYP has been widely demonstrated to cause cognitive disability. It mainly functions as a membrane protein of small synaptic vesicles in the central nerve system (Xiao et al., 2021). It directs the targeting of vesicle-specific membrane protein 2 (synaptobrevin) toward intracellular compartments (Chen et al., 2022). The other vital DEGs are SYT1, encoding synaptotagmin, integral membrane proteins of synaptic vesicles, and serves as a Ca^2+^ sensor in vesicular trafficking and exocytosis (Baker et al., 2018; Shi et al., 2020).

There are several canonical pathways in AD, including inflammatory responses, cholesterol, lipid metabolism, and endosomal vesicle recycling (Guerreiro and Hardy, 2014; Wang et al., 2021). Interestingly, metabolism and cellular biological functions were also observed in this study. It is noted that these DEGs were mainly enriched in exocytosis, synapse, and transport pathways in the GO database. Among them, the most enriched pathway in BPs is neurotransmitter transport and regulation of exocytosis. Apart from this, the most prominent pathway for enrichment in CCs is the presynapse and neuron projection terminus.

In contrast, protein kinase C binding and chloride transmembrane transporter activity are primarily significant pathways. Similarly, the most prominent enrichment in the KEGG database is the synapse, metabolism, and hormone-related pathways. All of the evidence mentioned above suggest that the DEGs between AD and controls are closely related to cell membrane function, transport, synapse, and metabolism.

Meanwhile, it is noted that AsymAD is a subtype of preclinical AD characterized as an asymptomatic at-risk state for AD, where beta-amyloids in the brain and CSF are thought to be the primary evidence (Mantzavinos and Alexiou, 2017). Basic scientists indicate that alterations in neurons, microglia, and astroglia drive the disease’s insidious progression before cognitive impairment appear (Elahi et al., 2020). In addition, data obtained through imaging studies with PiB-PET also suggests that Aβ deposits may appear up to two decades before the onset of clinical manifestations of dementia. There is growing evidence that pathological changes in AD have occurred 10 years before symptoms arise, but not all AsymADs will translate into SymADs. AsymADs are not the necessary stage of AD. Therefore, in the second part, we discussed the difference between AsymAD and SymAD to find the risk factors for converting AsymAD to SymAD and provide targets for subsequent drug treatment.

In the comparative analysis of AsymAD and SymAD, we obtained 19 genes by intersection. Among them, compared with AD genes through GeneCards^2^ and MalaCards^3^ databases, we found that CAP2, VSNL1, PAK1, SYP, ENC1, NEFM, ADCYAP1, SVOP, CHGB are related to Alzheimer’s disease; LOC387856 (CCDC184) and SYP are associated with dementia. Finally, according to logistic regression analysis, four molecules most related to clinical transformation (VSNL1, RTN1, FGF12, and ENC1) were screened out.

Visinin-like 1 (VSNL1) is a member of a subfamily of neuronal calcium sensor proteins. The encoded visinin-like protein 1 (VILIP-1) upregulates functional α4β2 nicotinic/acetylcholine receptors in hippocampal neurons (Recabarren and Alarcon, 2017). Several studies have shown that in patients with early symptomatic AD, the level of VILIP-1 in cerebrospinal fluid is closely related to whole and regional brain atrophy and is associated with amyloid load in normal individuals in cognition (Tarawneh et al., 2012). Moreover, VILIP-1 influences the intracellular neuronal signaling pathways involved in synaptic plasticity in the central nervous system. It also participates in cyclic nucleotide cascades, nicotinic signaling, and Ca^2+^ homeostasis, leading to neuronal loss (Groblewska et al., 2015). In this study, the expression of VSNL1 in SymAD was significantly downregulated, which was consistent with the results of Mirza and Rajeh (2017).

Reticulon 1 (RTN1) is an endoplasmic reticulum stress protein in the reticulon family involved in endocrine secretion and membrane trafficking. RTN1 is expressed predominantly in neuroendocrine tissues. Its function is mainly implicated in DNA binding or epigenetic modification, neuronal differentiation, and neurodegenerative diseases such as AD (Recabarren and Alarcon, 2017). Considerable evidence suggests that all RTN proteins and receptor NgR are engaged in the pathology change of AD by regulating the beta-site amyloid precursor protein-cleaving enzyme 1 (BACE1) function or APP processing, and thereby product amyloid β in the brain (Kulczynska-Przybik et al., 2021). Previous studies have observed a significant decrease in RTN1 expression in the frontal cortex of AD patients (Kim et al., 2000). Similarly, it was also significantly downregulated in the temporal cortex of symptomatic AD patients here.

The fibroblast growth factor 12 (FGF12) encodes the growth factor family protein involved in nervous system development and the positive regulation of voltage-gated sodium channel activity (Siekierska et al., 2016). FGF family members possess broad cell survival activities and are involved in various biological processes, including embryonic development, cell growth, morphogenesis, tissue repair, tumor growth, and invasion. The PI3K-Akt and apoptotic pathways in fibroblasts are the most relevant pathways to this gene. Gene Ontology annotations closely related FGF12 to growth factor activity and transmembrane transporter binding. This gene has been shown to play an essential role in the pathogenesis of epileptic encephalopathy (Deciphering Developmental Disorders Study, 2017).

The ectodermal-neural Cortex 1 (ENC1) gene is highly expressed in developing neurons and plays a role in the oxidative stress response as a regulator of the transcription factor Nrf2. This gene encodes a member of the kelch-related family of actin-binding proteins, which is implicated in neurite development and neuronal process formation during neuronal differentiation. In addition, ENC1 is upregulated *in vitro* models of neural injuries, such as oxygen-glucose deprivation or toxic intracellular protein aggregation and endoplasmic reticulum stress. Among its related pathways are the glucocorticoid receptor and wnt signaling pathway. Furthermore, based on cognitive, pathological, and genomic data, ENC1 was identified as a potential risk factor in cognitive performance and neuropathological burden in the aging population (White et al., 2017). Similar to other studies, we found a significant decrease in ENC1 expression in the temporal cortex of symptomatic AD patients (Wang et al., 2022).

In addition to the expression changes, the SNP loci of these genes are also worthy of further study. In previous researches, Hollingworth et al. (2012) found that VSNL1 (rs4038131) showed the most robust association with AD symptoms compared to controls. White et al. (2017) announced that rs76662990G locus in ENC1 was associated with slower cognitive decline in multiple domains. However, there is little evidence for the genetic locus associated with the risk of symptomatic AD, and more analysis and experimental studies are needed to gradually discover the mechanism behind it.

It is worth noting that our integrated dataset included a total of 184 patients with AD and 132 non-AD participants. The number of samples investigated ranged from 6 to 195 cases across the studies. There was no statistical difference among datasets in age. In the same way, we found that a total of 6 data sets obtained from the same platform (GPL10558) were included in the study of Zhang et al. (2019), of which the minimum sample size was 13, and the maximum was as many as 106. The three datasets we selected were also sequenced by using the same platform. Additionally, a total of 4 microarray data sets were included in the study of Yin et al. (2016). The small sample consisted of 22 cases of hepatocellular carcinoma and 21 liver tissue while the large sample covered 225 cases of hepatocellular carcinoma and 220 liver tissue. They also claimed that there was no significant difference between the datasets. We are trying to include as many datasets as possible. Sample size imbalance is inevitable as a consequence which is one of the drawbacks of such research. Researchers need to further think about how to solve this problem to improve the accuracy of analysis.

The result of the present study provides new insight into the earliest biological changes occurring in the brain before the manifestation of clinical AD symptoms. It offers new potential therapeutic targets for early disease intervention. VSNL1, RTN1, FGF12, and ENC1 may be the essential genes that progress asymptomatic AD to symptomatic AD. Moreover, they may serve as genetic risk factors to identify high-risk individuals showing an earlier onset of AD.

## Data availability statement

The original contributions presented in this study are included in this article/Supplementary material, further inquiries can be directed to the corresponding authors.

## Author contributions

JL, SY, and AX conceived and designed the study. YR and YM analyzed the data and composition of figures. WL and HL drafted the manuscript. All authors were involved in writing of the manuscript, gave final approval of the version, and agreed to be accountable for all aspects of the work.

## Footnotes

1
https://cn.string-db.org

2
https://www.genecards.org

3
https://www.malacards.org

## Supplementary material

Supplementary Figure 1 Data standardization and quality control. **(A,B)** The PCA graph before and after data set processing. **(C,D)** The density map before and after data set processing. **(E)** The box plot of comparison between AD group and control group after dataset processing.
