# Supplementary figures and images for: Identification of candidate genes associated with clinical onset of Alzheimer’s disease
Source: Front Neurosci. 2022 Dec 20;16:1060111. doi: 10.3389/fnins.2022.1060111 (PMC9808086; doi:10.3389/fnins.2022.1060111)

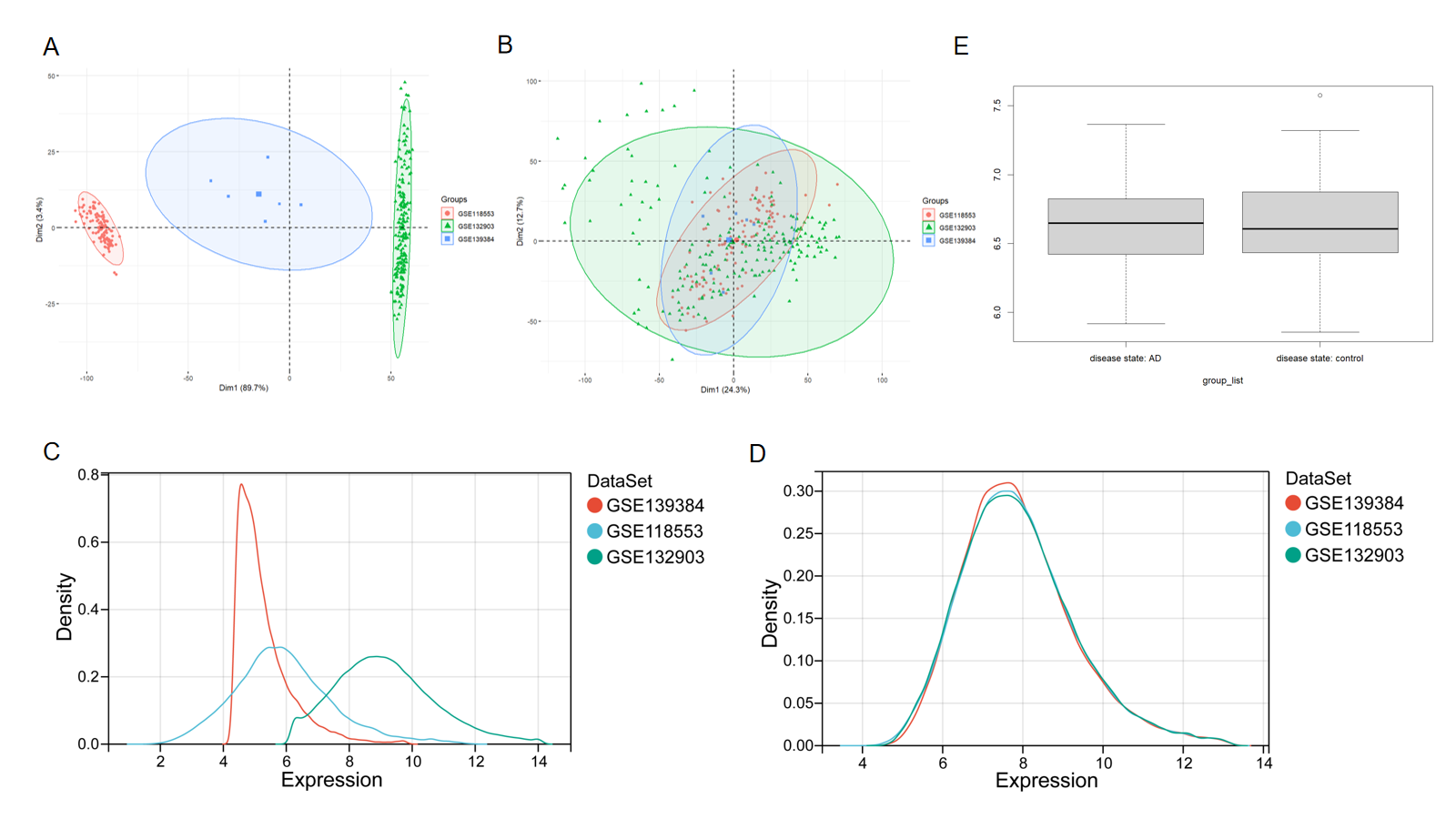

Supplement: Supplementary Figure 1 — Data standardization and quality control. (A,B) The PCA graph before and after data set processing. (C,D) The density map before and after data set processing. (E) The box plot of comparison between AD group and control group after dataset processing. [file Image_1.TIF]
